# Aqueous humor TGFβ and fibrillin-1 in Tsk mice reveal clues to POAG pathogenesis

**DOI:** 10.1038/s41598-024-53659-z

**Published:** 2024-02-12

**Authors:** James C. Tan, MinHee K. Ko, Jeong-Im Woo, Kenneth L. Lu, Jonathan A. Kelber

**Affiliations:** 1 Sightgene, Inc., 9227 Reseda Blvd, #182, Northridge, CA 91324-3137 USA; 2 Doheny Eye Institute, Pasadena, CA USA; 3 Department of Ophthalmology, University of California Los Angeles, Los Angeles, CA USA; 4 Developmental Oncogene Laboratory, California State University Northridge, Northridge, CA USA; 5 Department of Biology, Baylor University, Waco, TX USA

**Keywords:** Medical research, Molecular medicine, Pathogenesis

## Abstract

Aqueous humor (AH) and blood levels of transforming growth factor β (TGFβ) are elevated in idiopathic primary open angle glaucoma (POAG) representing a disease biomarker of unclear status and function. Tsk mice display a POAG phenotype and harbor a mutation of fibrillin-1, an important regulator of TGFβ bioavailability. AH TGFβ2 was higher in Tsk than wild-type (WT) mice (by 34%; p = 0.002; ELISA); similarly, AH TGFβ2 was higher in human POAG than controls (2.7-fold; p = 0.00005). As in POAG, TGFβ1 was elevated in Tsk serum (p = 0.01). Fibrillin-1 was detected in AH from POAG subjects and Tsk mice where both had similar levels relative to controls (p = 0.45). 350 kDa immunoblot bands representing WT full-length fibrillin-1 were present in human and mouse AH. A 418 kDa band representing mutant full-length fibrillin-1 was present only in Tsk mice. Lower molecular weight fibrillin-1 antibody-reactive bands were present in similar patterns in humans and mice. Certain bands (130 and 32 kDa) were elevated only in human POAG and Tsk mice (p ≤ 0.04 relative to controls) indicating discrete isoforms relevant to disease. In addition to sharing a phenotype, Tsk mice and human POAG subjects had common TGFβ and fibrillin-1 features in AH and also blood that are pertinent to understanding glaucoma pathogenesis.

## Introduction

The pathogenesis of primary open angle glaucoma (POAG)^1,2^, a leading cause of irreversible blindness worldwide, remains enigmatic. In POAG, levels and activity of transforming growth factor-β (TGFβ), a multifunctional growth factor, are elevated in the eye’s aqueous humor fluid (prominently TGFβ2)^3–10^ and blood (TGFβ1)^11^. The TGFβ anomaly represents a cryptic biomarker that has been reported in individuals with POAG across many ethnicities globally^3–10,12–14^. Its role in POAG pathogenesis and an interplay with major disease risk factors of age, intraocular pressure (IOP) and central corneal thickness (CCT)^15–19^ is unclear and worth trying to understand.

We recently reported^20^ that Tight skin (Tsk) fibrillin-1 mutant mice^21,22^ with systemically impaired tissue elasticity display a phenotype with defining features of human age-related POAG. These included age-related IOP elevation; fellow eye IOP asymmetry; IOP frequency distribution resembling human POAG; and relatively thin CCT. Major POAG risk factors were accompanied by an optic neuropathy evident as axonal attrition, age-related retinal ganglion cell decline with apoptosis, and visual deficit. Furthermore, levels and activity of aqueous humor TGFβ2, the prominently elevated human POAG aqueous humor TGFβ isoform^3,4,14^ were elevated. This raises an intriguing possibility that a fibrillin-1 or related defect contributes to the TGFβ anomaly and emergence of clinical and risk features of POAG.

Fibrillin-1 is a major extracellular matrix protein supporting elastic microfibrils and tissue elasticity^23,24^. It also serves as a reservoir for latent TGFβ by binding TGFβ to the extracellular matrix and regulating TGFβ bioavailability and activity. Fibrillin-1 mutation is associated with inappropriate TGFβ signaling, as seen in Marfan Syndrome^25–27^, an archetypal human condition due to fibrillin-1 mutation affecting eye, cardiovascular and bony systems. Marfan Syndrome patients have eyes with lens zonule friability causing ectopia lentis and increased prevalence of POAG^28^.

The eye’s aqueous humor drainage tissues such as trabecular meshwork and ciliary muscle are richly elastic and expansile tissues with abundant elastic microfibrils^29,30^. Elasticity of the trabecular meshwork supports a pulsatile dynamic of aqueous humor outflow and accommodates shifts in intraocular fluid volume that are integral to physiological IOP regulation^31–34^. In POAG, elastic microfibrils in the trabecular meshwork are degenerate^35^ and feature abnormal plaques containing fibrillin-1^29,30^. This is associated with a more rigid trabecular meshwork of diminished elasticity and mobility^31,36, 37^. Increased trabecular meshwork rigidity may be associated with elastic microfibrillar abnormality but also anomalous TGFβ that drives mesenchymal and profibrotic transition^38,39^. It is thus not surprising that aqueous humor outflow and IOP become dysregulated in POAG.

Phenotypes arising by fibrillin-1 mutation may overlap with those arising from mutations of related non-fibrillin proteins regulating elastic microfibrils^40–44^. For example, open angle glaucoma presentations are associated with mutations of fibrillin-1, ADAMTS10 (a disintegrin and metalloproteinase with thrombospondin motifs-10) and LTBP2 (latent transforming growth factor β binding protein-2) that are different proteins involved in regulating microfibrils^20,45–48^. LTBP2 mutation itself is linked with pseudoexfoliation syndrome, an elastopathy associated with polymorphisms of LOXL1 (lysl oxidase-like-1;^49^) playing roles in elastin maintenance and open angle glaucoma with elevated aqueous humor TGFβ^7,8, 14^. It could be that abnormalities of functionally related proteins supporting elastic microfibril homeostasis contribute to common disease pathways of which POAG is one. Hence characterizing Tsk mice may help us better understand not only potential contributions of fibrillin-1 mutation to POAG pathogenesis but also putatively related (but as yet unidentified) disorders contributing to glaucoma.

Our finding of POAG-like ocular clinical features and anomalous aqueous humor TGFβ in Tsk mice led us to search for biomarkers and clues to POAG pathogenesis in the mouse strain. TGFβ is typically sequestered to extracellular matrix-bound fibrillin-1 in tissues. But given the aqueous humor TGFβ anomaly of POAG and Tsk mice we wondered if fibrillin-1 might be present and perhaps also anomalous in aqueous humor in these conditions. We confirmed our earlier aqueous humor TGFβ2 observations^20^ in a larger population of Tsk mice then assayed aqueous humor fibrillin-1 in the mouse strain. For relevance, we performed parallel analyses of aqueous humor TGFβ2 and fibrillin-1 in human normal controls and individuals with POAG. Finally, we ascertained if aqueous humor findings have a systemic correlate by analyzing mouse blood levels of TGFβ and fibrillin-1. Our studies represent steps toward identifying biomarkers, understanding pathogenesis and informing on the extent to which Tsk mice model human POAG.

## Results

### Aqueous humor TGFβ2 is elevated in human POAG

We collected aqueous humor from (a) human POAG patients (n = 15) undergoing trabeculectomy surgery and (b) age-matched control subjects (n = 14) without a history of glaucoma undergoing cataract surgery. Patient demographics are shown in Table 1. Patients with POAG predominantly had advanced glaucomatous optic neuropathy with mean Humphrey visual field mean deviation of − 14.9 ± 9.9 dB (mean ± standard deviation) and cup-disc ratio of 0.9 ± 0.1.

**Table 1:** Demographic data of human POAG (n = 15) and normal control (n = 14) subjects contributing aqueous humor.

Diagnosis	No. subjects	Male (M)/Female (F)	Age (years)	CDR	Preop. IOP (mmHg)	No. IOP-lowering Meds	VF MD (dB)
POAG	15	9 M, 6F	68.9 ± 12.4	0.9 ± 0.1	17.2 ± 3.9	2.9 ± 0.8	-14.7 ± 9.9
Control	14	6 M, 8F	72.9 ± 31.2	0.4 ± 0.1	14.8 ± 2.9	0 (N/A)	N/A

CDR, cup/disc ratio; Preop. IOP, intraocular pressure before surgery (treated in POAG, untreated in controls); No. IOP-lowering meds, number of IOP-lowering medications before surgery; VF MD, visual field mean deviation. Column data for Age, CDR, Preop IOP, No. IOP-lowering Meds, VF MD reported as mean ± standard deviation (SD). Analysis of differences: age, p = 0.41; CDR, p = 1.8E−12; IOP, p = 0.01. Normal control subjects did not undergo visual field testing in the absence of glaucoma or a suspicion of glaucoma.

**Figure 1:**
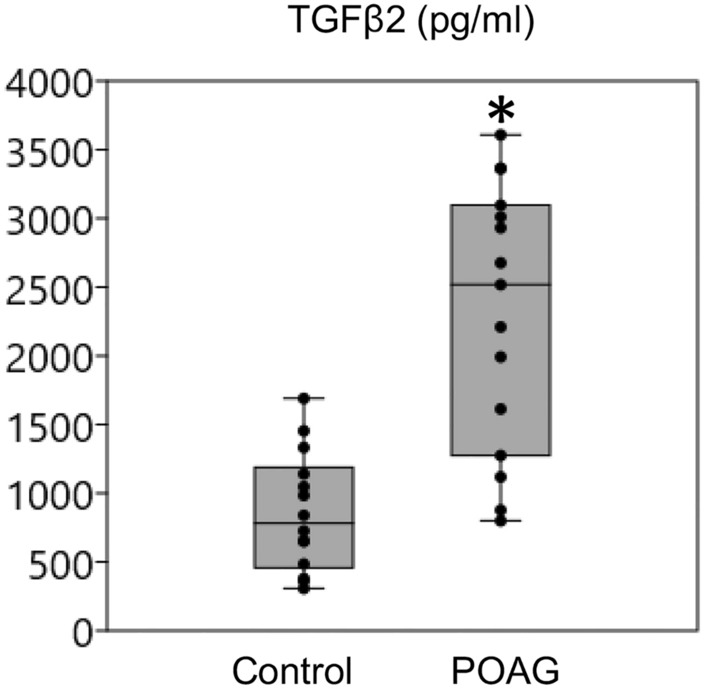
Total TGFβ2 levels in human aqueous humor (*p = 0.00005; ELISA; POAG, n = 15; control, n = 14). Box plots: 25th, 50th and 75th percentiles; error bars: 2.5th and 97.5th percentiles.

Aqueous humor total TGFβ2 levels in POAG eyes were elevated relative to normal control eyes (ELISA; Fig. 1). Aqueous humor TGFβ2 levels were a mean of 2.7-fold higher (p = 0.00005) in POAG (2297.1 ± 248.8 pg/ml; n = 15) compared with normal control eyes (860.6 ± 115.5 pg/ml).

The upper 97.5th percentile limit of aqueous humor TGFβ2 levels in normal control eyes was 1708 pg/ml. Two thirds (10/15; 67.7%) of POAG eyes had aqueous humor TGFβ2 levels exceeding this limit, equivalent to a 5% probability with a range of 2210–3608 pg/ml. Range of aqueous humor TGFβ2 levels in the remaining third of POAG eyes (5/15; 33.3%) was 877-1613 pg/ml, with TGFβ2 levels here indistinguishable from normal controls.

### Aqueous humor TGFβ2 is elevated in Tsk mice

**Figure 2:**
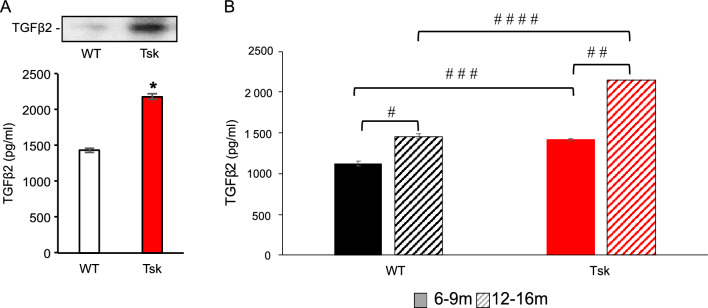
(**A**): Aqueous humor total TGFβ2 levels in Tsk (n = 20) and age-matched WT mice (n = 22) based on quantitative ELISA with immunoblot (top; cropped bands) confirmation (*p = 0.002; 5 independent experiments; age 10–15 months). See Supplementary data Fig. S1 for full blot from which TGFβ2 bands in 2A were cropped. (**B**) Aqueous humor total TGFβ2 levels in WT (black) and Tsk mice (red) aged 6–9 months (m) (solid) and 12-16 m (striped). n = 12–14 per group. ^#^p = 0.00005; ^##^p = 0.002: ^:###^p = 0.004; ^####^p = 0.005. Bars: mean. Error bars: standard error of mean.

Aqueous humor total TGFβ2 levels were 34.1% higher in Tsk mice aged 10–15 months (n = 20) than age-matched wild-type (WT) mice (n = 22) based on quantitative ELISA with immunoblot confirmation (p = 0.002; 5 independent experiments; Fig. 2A).

We checked for age-related changes and found higher aqueous humor TGFβ2 levels in Tsk mice aged 12–16 months (n = 14) than Tsk mice aged 6–9 months (p = 0.002; n = 12; 3 independent experiments; Fig. 2B). Aqueous humor TGFβ2 levels were also higher in WT mice aged 12–16 months (n = 12) than WT mice aged 6–9 months (p = 0.00005; n = 12; 3 independent experiments).

Aqueous humor total TGFβ2 levels were significantly higher in Tsk mice than age-matched WT mice in both age groups of 6–9 months (p = 0.004) and 12–16 months of age (p = 0.005; Fig. 2B). TGFβ2 aqueous humor levels were higher with age in both Tsk and WT mice but a higher rate of increase was seen in Tsk (63%) than WT mice (47%) over an equivalent age span.

### TGFβ2 RNA similar in WT and Tsk mouse anterior segment tissues

**Figure 3:**
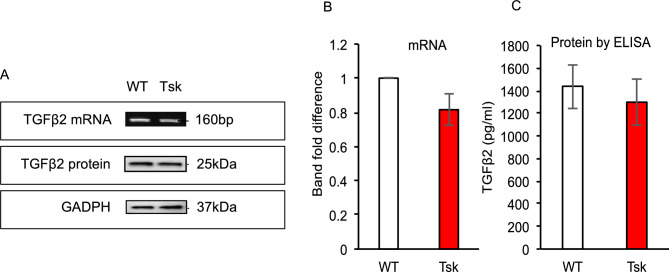
TGFβ2 total RNA and protein was extracted from mouse anterior segment tissues and analyzed by reverse-transcription PCR (**A**, top cropped bands), immunoblot (**A**, middle cropped bands; **B**, quantitative analysis), and ELISA (**C**, quantitative analysis). TGFβ2 transcript (**B**) and protein levels (**C**) in WT and Tsk mouse anterior segments tissues were similar (p = 0.5; 3 independent experiments). Bars: mean. Error bars: standard error of mean. See Supplementary data Fig. S2 (TGFβ2 mRNA), S3 (TGFβ2 protein) and S3 (GADPH) for full blots from which bands in 3A were cropped.

We analyzed mouse anterior segment tissues comprising cornea, sclera, iris, ciliary body and trabecular meshwork as a putative source of TGFβ in aqueous humor as shown in Fig. 3. Total TGFβ2 RNA extracted from the pooled mouse anterior segment tissues and analyzed by reverse-transcription PCR (RT-PCR) did not show significantly increased TGFβ2 RNA transcript levels in Tsk than WT mice (p = 0.5; total n = 12 each, three independent experiments; Fig. 3A, B). TGFβ2 protein levels were also not significantly different in mouse anterior segment tissues of Tsk and WT mice based on immunoblot and ELISA analysis (p = 0.5; total n = 12, three independent experiments; Fig. 3A, C).

### Aqueous humor fibrillin-1 similar with controls but discrete isoforms elevated in human POAG

**Figure 4:**
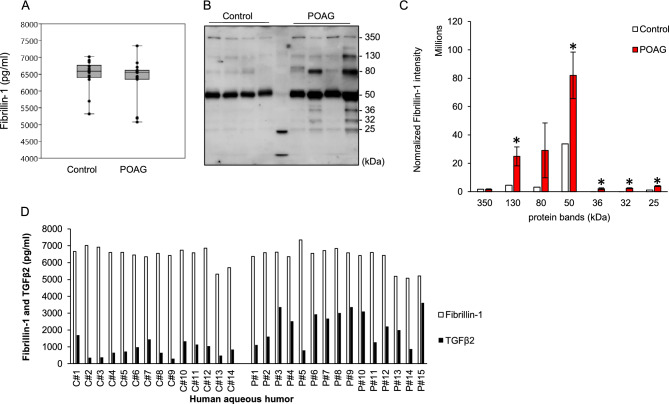
(**A**): Fibrillin-1 levels in human aqueous humor (p = 0.45; ELISA; POAG, n = 15; control, n = 14). (**B**): Representative immunoblots of human aqueous humor fibrillin-1 in POAG and normal controls. (**C**): Band densitometry of fibrillin-1 normalized to IgG negative controls (130 kDa, *p = 0.02; 50 kDa, *p = 0.04; 36 kDa, *p = 0.01; 32 kDa, *p = 0.001; 25 kDa, *p = 0.003; POAG, n = 15; control, n = 14). (**D**): Profiles of human aqueous humor fibrillin-1 levels relative to TGFβ2 levels in each POAG (P, n = 15) and normal control (C, n = 14) individual based on ELISA quantification.

Fibrillin-1 was present and detectable in the aqueous humor of POAG and normal control subjects by ELISA, as shown in Fig. 4. Aqueous humor fibrillin-1 levels were 6405.47 ± 595.29 pg/ml in normal control (n = 14) and 6483.46 ± 460.89 pg/ml in POAG (n = 15) eyes and not significantly different (p = 0.45; Fig. 4A) between the groups.

For fibrillin-1 immunoblot, we tested eight different anti-fibrillin-1 antibodies (Millipore MAB2502; LS Bio LS-c23555, LS-383476, LS-358981; Santa Cruz sc-20084; Abcam ab231094, ab24806; Thermo PA5-27358), some well-established, others based on selective affinities for different regions of the fibillin-1 molecule. Amongst these only the Abcam ab231094 anti-fibrillin-1 antibody revealed full-length fibrillin-1 in aqueous humor both in Tsk and WT mice and humans and only results derived by this antibody are reported here. It was important to identify full-length fibrillin-1 of the target molecular weight (350 kDa) to establish a reference for the presence of fibrillin-1 in aqueous humor and loading controls for standardization.

Our quantitative analysis utilized normalized conditions based on equal protein loading in lanes for SDS-PAGE as quantified by Bradford assay; further confirmation/adjustment by Ponceau S staining after membrane transfer; and loading controls. Figure 4B is a qualitative depiction of representative examples. Figure 4C aggregates analyzed samples and quantifies differences and variation in bands of note with reference to standardized loading controls comprising full-length fibrillin-1.

Immunoblot analysis of fibrillin-1 in human aqueous humor showed discrete bands at 350 kDa, 130 kDa, 80 kDa, 50 kDa, 36 kDa, 32 kDa and 25 kDa (Fig. 4B). Densitometry showed similar densities of the 350 kDa band representing full-length fibrillin-1 in POAG and normal control aqueous humor (p = 0.97; Fig. 4C). Many lower molecular weight bands (130 kDa, 50 kDa, 36 kDa, 32 kDa and 25 kDa) were significantly denser in POAG than normal control aqueous humor, however (Fig. 4C, asterisks; all differences p < 0.04). 32 ka and 36 kDa bands were present in POAG but virtually undetectable in normal controls.

Aqueous humor fibrillin-1 and TGFβ2 levels in human POAG and normal control individuals were profiled based on quantitative ELISA, as shown in Fig. 4D. Human aqueous humor fibrillin-1 levels were not correlated with TGFβ2 levels in POAG (R^2^ = 9E−05) or normal control individuals (R^2^ = 0.0085; scatter plots not shown).

### Aqueous humor fibrillin-1 similar with WT but discrete isoforms elevated in Tsk mice

**Figure 5:**
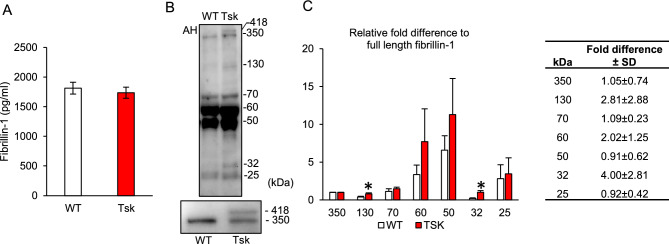
(**A**): Fibrillin-1 levels in mouse aqueous humor (p = 0.8; ELISA). (**B**): Immunoblot analysis of mouse aqueous humor showing full-length fibrillin-1 and lower molecular weight bands (representative of 4 experiments, total n = 40 mice). Inset (bottom): magnified view of full-length bands. (**C**): Fold-densitometry difference of fibrillin-1 fragments relative to full-length fibrillin-1 (shown as 350kD). *p = 0.04 for 130 kDa; *p = 0.02 for 32 kDa; 4 experiments. Bars: mean. Error bars: standard error of mean. Table: Tsk:WT mouse fold-difference of fibrillin-1 band densitometry in aqueous humor (mean ± standard deviation (SD), 4 experiments).

Fibrillin-1 was present and detectable in the aqueous humor of Tsk and WT mice by ELISA analysis, as shown in Fig. 5. Aqueous humor fibrillin-1 levels in Tsk mice aged 10–15 months and age-matched WT mice were not significantly different (p = 0.8; n = 20 per group; both aged 10-15 m; Fig. 5A).

Immunoblot analysis of fibrillin-1 in WT and Tsk mouse aqueous humor showed discrete bands representing WT full-length fibrillin-1 (350 kDa; Fig. 5). A 418 kDa band representing the mutant full-length fibrillin-1 protein known to co-exist with WT full-length fibrillin-1 in Tsk mouse tissues was present in aqueous humor from Tsk but not WT mice. Additional discrete bands of lower molecular weight were present at 130 kDa, 70 kDa, 60 kDa, 50 kDa, 32 kDa and 25 kDa.

Densitometry showed similar band densities at 350-418 kDa (full-length fibrillin-1), 70 kDa, 60 kDa, 50 kDa and 25 kDa in Tsk and WT mouse aqueous humor (all p > 0.05 for analysis of differences; 4 independent experiments; Fig. 5C). But band densities at 130 kDa (2.8-fold; p = 0.04) and 32 kDa (fourfold; p = 0.02) were significantly higher in Tsk than WT mice (Fig. 5C and table).

### Fibrillin-1 detected and TGFβ1 elevated in Tsk mouse serum

**Figure 6:**
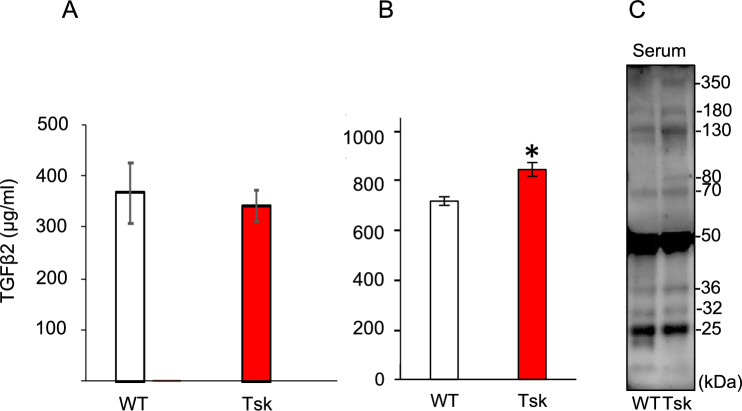
Serum total TGFβ2 (**A**; p = 0.32) and TGFβ1 (**B**; *p = 0.01) levels in WT and Tsk mice based on quantitative ELISA. (**C**): Representative immunoblot analysis of fibrillin-1 in WT and Tsk mouse serum.

We analyzed mouse serum to determine if TGFβ and fibrillin-1 observations in aqueous humor are also seen systemically as reported in human POAG^11^. Serum total TGFβ1 levels were significantly higher in Tsk than WT mice (p = 0.01; n = 10/group; aged 3–4 months; Fig. 6B). Serum total TGFβ2 levels were not significantly different between Tsk and WT mice (p = 0.32; n = 5/group; Fig. 6A).

Immunoblot of fibrillin-1 in WT and Tsk mouse serum showed 350 kDa, 180 kDa, 130 kDa, 80 kDa, 70 kDa, 50 kDa, 36 kDa, 32 kDa, and 25 kDa bands (Fig. 6C; representative of 10 samples), resembling the fibrillin-1 band profile of aqueous humor, with the exception that the WT full-length 350 kDa band was less dense and Tsk full-length 418 kDa band was not clearly discernable in mutants.

## Discussion

Aqueous humor TGFβ2 levels were 2.7-fold higher in human POAG subjects than normal controls, within the reported human POAG:control range of 1.51–2.75^4^. Two thirds of individuals with POAG showed elevated aqueous humor total TGFβ2 while a remaining third had aqueous humor TGFβ2 levels that were indistinguishable from normal control levels. Hence while the majority of our POAG subjects—all with advanced disease—showed elevated aqueous humor TGFβ2, this biomarker was not universally present. This suggests aqueous humor TGFβ2 levels per se do not serve as a universal biomarker of POAG, perhaps reflecting the heterogenous nature of the condition.

We confirmed our previous observation of elevated aqueous humor total TGFβ2 in larger samples of Tsk mice displaying features of POAG^20^. Aqueous humor TGFβ2 levels were a third higher in Tsk than WT mice and levels rose with age in both strains, but more so in Tsk mice. Elevated aqueous humor TGFβ activity is well documented in human POAG^3–8,10^ and we have reported the same in Tsk mice^20^, wherein the level of active TGFβ2 in Tsk mouse aqueous humor was over three-fold higher compared with WT mice^20^. The finding of elevated serum TGFβ1 in Tsk mice also mirrors observations in human POAG^11^, further supporting the notion that Tsk mice model human POAG. The presence of anomalous aqueous humor and hematogenous TGFβ places Tsk mouse ocular findings within a context of systemic disease from an identifiable common mutation. To our best knowledge a similar scenario is not known for common human POAG but our observations suggest it is worth keeping an open mind to this possibility.

Our studies of Tsk mouse anterior segment tissues did not implicate heightened TGFβ2 RNA synthesis as a source of increased TGFβ2 in mutant aqueous humor. Our reverse transcription PCR studies were based in tissue that was postmortem and pooled in which timing of harvesting was not standardized and some adjustment of conditions may be necessary for real-time PCR in future studies. An alternative source of elevated TGFβ to consider is the fibrillin-1-bound TGFβ repository in anterior segment tissues where extracellular matrix perturbation might inordinately affect TGFβ levels and activity in fluids surrounding tissues. This latter notion seems plausible given the fibrillin-1 mutation^21^, microfibril structural abnormalities^22,50,51^ and expected fibrillin-1 dysfunction^23–28,40^ in Tsk mice.

We did not find a significant difference in aqueous humor fibrillin-1 levels between Tsk and WT mice or between human POAG and normal controls with ELISA-based quantification. Immunoblot densitometric comparisons of full-length fibrillin-1 in the aqueous humor of Tsk and WT mouse eyes and human POAG and normal control eyes also did not show significant differences. Additionally, we did not find significant correlation between aqueous humor fibrillin-1 levels and TGFβ2 levels. Our analyses of full-length fibrillin-1 in aqueous humor thus did not specifically explain the aqueous humor TGFβ2 anomaly in human POAG or Tsk mouse eyes.

Full-length fibrillin-1 is typically extracellular matrix-bound in tissues^23,24^ and it was surprising to find the full-length protein in aqueous humor fluid and serum. The same observation was true for human and mouse aqueous humor. This suggests that a certain proportion of full-length fibrillin-1 enters the aqueous humor in soluble form under physiologic conditions, the purpose of which is unclear. By the same token, mutant full-length fibrillin-1, which is present in Tsk but not WT mouse tissues^50,51^, was only identified in Tsk mouse aqueous humor. Similarly, full-length wild-type fibrillin-1 was identified in Tsk and WT mouse serum, albeit less prominently perhaps due to serum factors promoting protein degradation. It is not surprising that Tsk mutant full-length fibrillin-1 in serum was even harder to detect as the mutant protein may be relatively unstable and susceptible to degradation^52–55^. Fibrillin-1 binds TGFβ in the extracellular matrix where it regulates TGFβ bioavailability and activity. It is conceivable that fibrillin-1 abnormality affects the stability of its associated extracellular matrix-bound TGFβ repository, causing dysregulation and release of TGFβ into surrounding fluids such as aqueous humor or blood. Alternatively, given the presence of fibrillin-1 in soluble form, perhaps some (as yet unknown) association between fibrillin-1-TGFβ also exists in fluid phase; and if so, fibrillin-1 abnormality could contribute to aqueous humor TGFβ anomalies of the type seen in Tsk mice. These possibilities are relevant to POAG and worth exploring in future studies.

The aqueous humor pattern of lower molecular weight immunoblot bands was similar in humans and mice. Certain bands had significantly higher densities in human POAG and Tsk mice compared with their respective controls and certain denser bands (e.g., 130 kDa and 32 kDa) were common to human POAG and Tsk mice. The latter indicates discrete isoforms relevant to disease that we are characterizing further in separate studies. Certain lower molecular weight bands may represent fibrillin-1 digestion products arising by extracellular matrix turnover and proteolytic degradation^52–55^. For example, collagenase digestion of fibrillin-1 yields prominent fragments around 30–40 kD and 80 kD^54^; while matrix metalloproteinase digestion yields bands around 40–50 kD and 70–90 kD^52,53^; both ranges encompass the lower molecular weight bands we identified. It is also worth considering that certain fibrillin-1 fragment anomalies are associated with vascular abnormalities such as aortic dissection^56–58^, which itself is a prominent feature of Marfan Syndrome from fibrillin-1 mutations.

As with our prior phenotypic observations^20^, we report that Tsk mice and human individuals with POAG share common TGFβ anomalies and fibrillin-1 features in the intraocular and intravascular fluids. These represent clues to primary glaucoma pathogenesis that we believe are worthy of further attention.

## Methods

### Aqueous humor collection

Human subjects were classified as (a) having POAG, or (b) normal controls based on history, clinical examination and visual field testing by Humphrey 24–2 automated perimetry (Carl Zeiss Meditec) and optic disc photography. Subjects with POAG had characteristic cupping of the optic disc associated with corresponding visual field defects. POAG subjects underwent trabeculectomy for inadequately controlled IOP according to standard of care indications. All were using IOP-lowering medication and were free of other eye disease apart from glaucoma and cataract. Normal control subject eyes were free of other eye disease apart from cataract; did not take ocular medications; and indication for surgery was visually significant cataract. All subjects gave informed consent before aqueous humor was collected during trabeculectomy (POAG) or cataract surgery (normal controls).

Aqueous humor was collected and pooled (typically 30–100 μl) from both eyes of anesthetized mice using anterior chamber perfusion apparatus under a dissecting microscope as we have previously described in detail^59,60^. Briefly, a 35-gauge needle (5 mm long, Medicom, Canada) connected to Hamilton syringe (10 µl with luer tips) on a micromanipulator (MM33 Rechts, Germany) was used to cannulate the anterior chamber through the peripheral cornea near the limbus without disrupting adjacent tissues. The syringe was capped and placed on ice before transportation to the laboratory. Each aqueous humor sample was centrifuged (4 °C, 13,000 rpm, 5 min) to remove possible cells and debris. The supernatant was stored in a microcentrifuge tube at − 80 °C. Samples meeting above criteria and volume requirements were randomly selected for analysis based on diagnosis and without knowledge of patient identity or past ocular history.

### Animal husbandry and anesthesia

Tsk mice (B6.Cg-Fbn1Tsk/J. stock #014632^21^ and C57BL/6J (stock # 00064) were purchased (The Jackson Laboratory) and bred in-house. Tsk homozygotes are embryonically lethal and we used Tsk heterozygote mice for our study. Tsk mice and wild-type (WT) littermates as a control from the colony were used 3–15 months (m) of age. Both females and males were used in all experiments. Inter-strain comparisons involved age-matched mice. The mice were raised and housed in air-filtered clear cages with a bedding of pine shavings, subject to a 12 h light/dark cycle, and fed ad libitum. Mice were anesthetized with a mixture of ketamine (85 mg/Kg, Ketaject, Phoenix Pharmaceutical, Inc.), xylazine (8.5 mg/Kg, AnaSed; Lloyd Laboratories) and acepromazine (2.125 mg/Kg, Boehringer Ingelheim), injected intraperitoneally. Anesthesia was titrated to achieve a depth permitting aqueous humor collection. One drop of topical proparacaine hydrochloride ophthalmic solution (0.5%, Akorn Inc.) was applied to the cornea prior to experiments requiring ocular surface contact. Mice were rested on a warming platform or under a heating blanket to maintain body temperature during experiments.

### Genotyping Tsk mice by standard PCR

Tail tips (~ 1 mm) of each new pup were collected after weaning and genotyping was performed using standard PCR methods as recommended (The Jackson Laboratory). Briefly, three primers were synthesized (Integrated DNA Technologies Inc): 5′GGC TCC TTC CTC CCA CTT AG 3′ (WT); 5′ATC CCT GGG ACC ATA ACA CA 3′ (Common); 5′GAG TCC GAG TGT CCC TCA AG 3′ (Mutant). PCR was performed in a Mastercycler® (Eppendorf) using a protocol of 1 cycle of 94 °C for 2 min (min), 28 cycles of 94 °C for 15 s, 60 °C for 15 s, and 72 °C for 10 s. Tsk heterozygotes (173 and 278 base-pair (bp)) and WT littermates (278 bp) were identified following 2% agarose gel electrophoresis.

### Mouse aqueous humor and serum preparation

Aqueous humor was collected from both eyes of each anesthetized mouse using anterior chamber perfusion apparatus under a dissecting microscope^60^. Briefly, a 35-gauge needle (5 mm long, Medicom, Canada) connected to Hamilton syringe (10 µl with luer tips) on a micromanipulator (MM33 Rechts, Germany) was used to cannulate the anterior chamber through the cornea without disrupting adjacent tissues. Mouse blood was obtained from the tail vein (~ 100 µl per mouse, n = 10 each group) and serum was isolated after centrifugation. Total protein was quantified (BCA protein assay kit, Thermo Scientific) in aqueous humor and serum for ELISA and immunoblot analysis.

### ELISA of aqueous humor and blood

Total TGFβ1, TGFβ2, or fibrillin-1 levels were measured in human aqueous humor and mouse aqueous humor and serum (human aqueous humor, n = 14–15 per group; Tsk and age-matched WT mice, ages 6-9 m and 10-15 m; n = 20–22 mice per group) using a Mouse/Rat/Canine/Porcine TGFβ1 (Cat# MB100B, R&D Systems), TGFβ2 (mouse, Cat# MB200, R&D Systems; Human, Cat# ab100648, Abcam), fibrillin-1 (mouse, Cat# LS-F24166; Human, Cat# LS-F4203, LifeSpan BioSciences). Each standard protein was serially diluted in triplicates. Samples in triplicate were prepared from two independent sets of pooled aqueous humor or individual serum. TGFβ in the samples was activated with 1N HCl (1:2 dilution; 10 min at room temperature (RT)) then neutralized with 1.2N NaOH/0.5M HEPES according to the manufacturer’s protocol.

### Immunoblot analysis

Pooled aqueous humor from WT and Tsk mice (n = 10–12 mice/group; 5 independent experiments) were used for immunoblotting. For immunoblotting, 10-30 µl of sample was heated for 5 min at 95 °C with reduced 6× Laemmli SDS sample buffer (Bioland Scientific LLC) and run in SurePAGE, 4–12% Bis–Tris gel (GeneScript). Immunoblot samples were loaded in equal protein concentrations for SDS-PAGE based on Bradford assays. The proteins were transferred onto PVDF membranes (Bio-Rad) and stained with Ponceau S staining solution (Bio-Rad) to verify transfer efficiency. Membranes were washed in Tris-buffered saline plus 0.1% Tween 20 (TBST) and blocked using 5% nonfat dry milk in TBST for 1 h at RT then incubated with primary antibodies (fibrillin-1 (ab231094, Abcam) or TGFβ2 (ab36495, Abcam)) overnight at 4 °C. After washing 3 times, membranes were incubated for 1 h at RT with HRP-conjugated secondary antibody (ab6789 or ab6721, Abcam) in blocking solution. Washed membranes were then incubated with a SuperSignal substrate (Thermo Scientific) for 1 min at RT and signal detection and densitometry analysis were performed with BioRad ChemiDoc XRS system.

### Reverse transcription PCR

Total RNA was isolated from mouse anterior segment tissue (n = 6 mice/group; age 6-9 m; 2 independent experiments) using an RNA isolation kit (Qiagen). First strand cDNA was synthesized from 2 µg of RNA using a High capacity cDNA reverse transcription kit (Applied Biosystems). PCR was performed for TGFβ2 and glyceraldehyde 3-phosphate dehydrogenase (GAPDH) as a housekeeping control for normalization. PCR products were analyzed in 2% agarose and band intensities were analyzed using a BioRad ChemiDoc XRS system. PCR primers were synthesized (Integrated DNA Technologies Inc.): mouse TGFβ2 (5′ CAG GAG TGG CTT CAC CAC AAA G 3′, 5′ TGG CAT ATG TAG AGG TGC CAT CA 3′), mouse GAPDH (5′ AAG CCC ATC TTC CA 3′, 5′ CCT GCT TCA CCA CCT TCT TG 3′). PCR was performed in a Mastercycler® Thermal Cycler (Eppendorf): 1 cycle of 94 °C for 4 min; 35 cycles of 94 °C for 1 min; 57 ~ 60 °C for 1 min and 72 °C for 1 min; and extension cycle of 72 °C for 10 min.

### Albumin/IgG depletion

Serum and aqueous humor may contain albumin and IgG, which can interfere with antibody sensitivity and specificity. To mitigate this potential interference, each sample was diluted with binding/washing buffer based on he estimated binding capacity of 4 µg of albumin and IgG per sample per microliter of gel slurry using a Pierce™ Albumin/IgG Removal Kit (Thermo Scientific, Cat #89875). To assess the efficiency of the albumin/IgG removal process, both eluted and gel slurry fractions were analyzed by immunoblot.

## Statistics

Hypothesis testing was performed using two-tailed Students t-tests unless otherwise specified. The Mann–Whitney test was used when data distribution was non-parametric. Results were presented in the text as the mean ± standard deviation (SD). Data was presented in graphs as the mean with error bars for standard error of mean (SEM). Boxplots showed the median, 25th and 75th centiles and error bars for 2.5th and 97.5th centiles. Statistical analysis and graphing were performed in software packages, Past 3.0 (Paleontological Statistics Software Package; University of Oslo, Norway) and Excel for Mac 2011 software (Microsoft Corp). Asterisks (*) in graphs indicated p ≤ 0.05 representing statistical significance, with exact p-values noted in the corresponding figure legend.

### Study approval

All research on human subjects adhered to the tenets of the Declaration of Helsinki and received institutional review board (IRB) approval (UCLA IRB Protocol#16-001433) before collection of aqueous humor. All methods were carried out in accordance with relevant guidelines and regulations. These complied with the ARVO Statement for Use of Animals in Ophthalmic and Vision Research and were reported in accordance with ARRIVE guidelines. Approval for animal studies had been obtained from Institutional Animal Care and Use Committees (IACUC) at University of California, Los Angeles (2014-089-03A) and California State University Northridge (#1920-005c).

### Supplementary Information

Supplementary Figures.

## Data Availability

The datasets used and/or analyzed and generated during the current study are available from the corresponding author (JCT) on reasonable request.

## References

[1] Tan JC, Kaufman PL, Yanoff M, Duker JS (2023). Primary open angle glaucoma. Yanoff & Duker's Ophthalmology.

[2] Weinreb RN, Khaw PT (2004). Primary open-angle glaucoma. Lancet.

[3] Tripathi RC (1994). Aqueous humor in glaucomatous eyes contains an increased level of TGF-beta 2. Exp. Eye Res..

[4] Agarwal P, Daher AM, Agarwal R (2015). Aqueous humor TGF-beta2 levels in patients with open-angle glaucoma: A meta-analysis. Mol. Vis..

[5] Inatani M (2001). Transforming growth factor-beta 2 levels in aqueous humor of glaucomatous eyes. Graefes Arch. Clin. Exp. Ophthalmol..

[6] Ochiai Y, Ochiai H (2002). Higher concentration of transforming growth factor-beta in aqueous humor of glaucomatous eyes and diabetic eyes. Jpn. J. Ophthalmol..

[7] Picht G, Welge-Luessen U, Grehn F, Lütjen-Drecoll E (2001). Transforming growth factor beta 2 levels in the aqueous humor in different types of glaucoma and the relation to filtering bleb development. Graefes Arch. Clin. Exp. Ophthalmol..

[8] Ozcan AA, Ozdemir N, Canataroglu A (2004). The aqueous levels of TGF- 2 in patients with glaucoma. Int. Ophthalmol..

[9] Yamamoto N, Itonaga K, Marunouchi T, Majima K (2005). Concentration of transforming growth factor beta2 in aqueous humor. Ophthalm. Res..

[10] Min SH, Lee TI, Chung YS, Kim HK (2006). Transforming growth factor-beta levels in human aqueous humor of glaucomatous, diabetic and uveitic eyes. Korean J. Ophthalmol..

[11] Kuchtey J (2014). Elevated transforming growth factor beta1 in plasma of primary open- angle glaucoma patients. Invest. Ophthalmol. Vis. Sci..

[12] Trivedi RH, Nutaitis M, Vroman D, Crosson CE (2011). Influence of race and age on aqueous humor levels of transforming growth factor-beta 2 in glaucomatous and nonglaucomatous eyes. J. Ocul. Pharmacol. Ther..

[13] Stefan C (2008). TGF-beta2 involvements in open angle glaucoma (Romanian). Oftalmologia.

[14] Takai Y, Tanito M, Ohira A (2012). Multiplex cytokine analysis of aqueous humor in eyes with primary open-angle glaucoma, exfoliation glaucoma, and cataract. Invest. Ophthalmol. Vis. Sci..

[15] Brandt JD, Beiser JA, Kass MA, Gordon MO (2001). Central corneal thickness in the ocular hypertension treatment study (OHTS). Ophthalmology.

[16] Gordon MO (2002). The ocular hypertension treatment study: Baseline factors that predict the onset of primary open-angle glaucoma. Arch. Ophthalmol..

[17] Wilson RM, Martone JF, Ritch R (1996). Epidemiology of chronic open-angle glaucoma. The Glaucomas.

[18] Lee AJ, Rochtchina E, Mitchell P (2004). Intraocular pressure asymmetry and undiagnosed open-angle glaucoma in an older population. Am. J. Ophthalmol..

[19] Mitchell P, Smith W, Attebo K, Healey PR (1996). Prevalence of open-angle glaucoma in Australia. The Blue Mountains Eye Study. Ophthalmology.

[20] Ko MK (2022). Fibrillin-1 mutant mouse captures defining features of human primary open glaucoma including anomalous aqueous humor TGF beta-2. Sci. Rep..

[21] Siracusa LD (1996). A tandem duplication within the fibrillin 1 gene is associated with the mouse tight skin mutation. Genome Res..

[22] Akita M, Lee SH, Kaneko K (1992). Electron microscopic observations of elastic fibres in the lung and aorta of tight-skin and beta-aminopropionitrile-fed mice. Histol. Histopathol..

[23] Handford PA, Downing AK, Reinhardt DP, Sakai LY (2000). Fibrillin: From domain structure to supramolecular assembly. Matrix Biol..

[24] Ramirez F, Rifkin DB (2009). Extracellular microfibrils: Contextual platforms for TGFbeta and BMP signaling. Curr. Opin. Cell Biol..

[25] Sakai LY, Keene DR, Renard M, De Backer J (2016). FBN1: The disease-causing gene for Marfan syndrome and other genetic disorders. Gene.

[26] Neptune E (2003). Dysregulation of TGF-beta activation contributes to pathogenesis in Marfan syndrome. Nat. Genet..

[27] Habashi JP (2006). Losartan, an AT1 antagonist, prevents aortic aneurysm in a mouse model of Marfan syndrome. Science.

[28] Izquierdo NJ, Traboulsi EI, Enger C, Maumenee IH (1992). Glaucoma in the Marfan syndrome. Trans. Am. Ophthalmol. Soc..

[29] Rohen JW, Lutjen-Drecoll E, Flugel C, Meyer M, Grierson I (1993). Ultrastructure of the trabecular meshwork in untreated cases of primary open-angle glaucoma (POAG). Exp. Eye Res..

[30] Ueda J, Yue BY (2003). Distribution of myocilin and extracellular matrix components in the corneoscleral meshwork of human eyes. Invest. Ophthalmol. Vis. Sci..

[31] Johnstone M, Martin E, Jamil A (2011). Pulsatile flow into the aqueous veins: Manifestations in normal and glaucomatous eyes. Exp. Eye Res..

[32] Johnstone MA (2004). The aqueous outflow system as a mechanical pump: Evidence from examination of tissue and aqueous movement in human and non-human primates. J. Glaucoma.

[33] Khatib TZ (2019). Hemoglobin video imaging provides novel in vivo high-resolution imaging and quantification of human aqueous outflow in patients with glaucoma. Ophthalmol. Glaucoma.

[34] Huang AS (2017). Aqueous angiography in living nonhuman primates shows segmental, pulsatile, and dynamic angiographic aqueous humor outflow. Ophthalmology.

[35] Chi HH, Katzin HM, Teng CC (1957). Primary degeneration in the vicinity of the chamber angle; as an etiologic factor in wide-angle glaucoma: II. Am. J. Ophthalmol..

[36] Last JA (2011). Elastic modulus determination of normal and glaucomatous human trabecular meshwork. Invest. Ophthalmol. Vis. Sci..

[37] Johnstone M, Xin C, Tan J, Martin E, Wen J, Wang RK (2021). Aqueous outflow regulation—21st century concepts. Prog. Retin. Eye Res..

[38] Gottanka J, Chan D, Eichhorn M, Lütjen-Drecoll E, Ethier CR (2004). Effects of TGF-beta2 in perfused human eyes. Invest. Ophthalmol. Vis. Sci..

[39] Nakamura Y (2002). Signaling mechanism of TGF-beta1-induced collagen contraction mediated by bovine trabecular meshwork cells. Invest. Ophthalmol. Vis. Sci..

[40] Sengle G (2012). Microenvironmental regulation by fibrillin-1. PLoS Genet..

[41] Faivre L (2003). Clinical homogeneity and genetic heterogeneity in Weill-Marchesani syndrome. Am. J. Med. Genet.

[42] Haji-Seyed-Javadi R (2012). LTBP2 mutations cause Weill-Marchesani and Weill-Marchesani-like syndrome and affect disruptions in the extracellular matrix. Hum. Mutat..

[43] Morales J (2009). Homozygous mutations in ADAMTS10 and ADAMTS17 cause lenticular myopia, ectopia lentis, glaucoma, spherophakia, and short stature. Am. J. Hum. Genet..

[44] Ahram D (2009). A homozygous mutation in ADAMTSL4 causes autosomal-recessive isolated ectopia lentis. Am. J. Hum. Genet..

[45] Kuchtey J (2013). Screening ADAMTS10 in dog populations supports Gly661Arg as the glaucoma-causing variant in beagles. Invest. Ophthalmol. Vis. Sci..

[46] Kuchtey J (2011). Mapping of the disease locus and identification of ADAMTS10 as a candidate gene in a canine model of primary open angle glaucoma. PLoS Genet..

[47] Jelodari-Mamaghani S (2013). Contribution of the latent transforming growth factor-beta binding protein 2 gene to etiology of primary open angle glaucoma and pseudoexfoliation syndrome. Mol. Vis..

[48] Saeedi O (2018). Delineation of novel compound heterozygous variants in LTBP2 associated with juvenile open angle glaucoma. Genes.

[49] Thorleifsson G (2007). Common sequence variants in the LOXL1 gene confer susceptibility to exfoliation glaucoma. Science.

[50] Kielty CM (1998). The Tight skin mouse: Demonstration of mutant fibrillin-1 production and assembly into abnormal microfibrils. J. Cell Biol..

[51] Gayraud B, Keene DR, Sakai LY, Ramirez F (2000). New insights into the assembly of extracellular microfibrils from the analysis of the fibrillin 1 mutation in the tight skin mouse. J. Cell Biol..

[52] Ashworth JL (1999). Fibrillin degradation by matrix metalloproteinases: Implications for connective tissue remodelling. Biochem. J..

[53] Hindson VJ (1999). Fibrillin degradation by matrix metalloproteinases: Identification of amino- and carboxy-terminal cleavage sites. FEBS Lett..

[54] Kuo CL (2007). Effects of fibrillin-1 degradation on microfibril ultrastructure. J. Biol. Chem..

[55] Murai C (1998). Spontaneous occurrence of anti-fibrillin-1 autoantibodies in tight-skin mice. Autoimmunity.

[56] Carlson EJ (2022). Circulating fibrillin fragment concentrations in patients with and without aortic pathology. JVS Vasc. Sci..

[57] Marshall LM (2013). Thoracic aortic aneurysm frequency and dissection are associated with fibrillin-1 fragment concentrations in circulation. Circ. Res..

[58] Charbonneau NL (2010). In vivo studies of mutant fibrillin-1 microfibrils. J Biol. Chem..

[59] Ko MK, Kim EK, Gonzalez JM, Tan JC (2016). Dose- and time-dependent effects of actomyosin inhibition on live mouse outflow resistance and aqueous drainage tissues. Sci. Rep..

[60] Ko MK, Yelenskiy A, Gonzalez JM, Tan JC (2014). Feedback-controlled constant-pressure anterior chamber perfusion in live mice. Mol. Vis..

