# Supplementary material for: Aqueous humor TGFβ and fibrillin-1 in Tsk mice reveal clues to POAG pathogenesis
Source: Sci Rep. 2024 Feb 12;14:3517. doi: 10.1038/s41598-024-53659-z (PMC10861487; doi:10.1038/s41598-024-53659-z)
Supplement: Supplementary file 1 — Supplementary Figures. [file 41598_2024_53659_MOESM1_ESM.docx]

**SUPPLEMENTARY INFORMATION**

Control POAG

**Fig S5.** **Human aqueous humor fibrillin-1.** Full blot from which

Fig. 4B immunoblot of Fibrillin-1 in human normal control and POAG

aqueous humor was cropped. White enclosure: cropped bands shown

in Fig. 4B.

WT Tsk

**Fig S6. Fibrillin-1 in mouse aqueous humor.** Full blot from which Fig. 5B

immunoblot of Fibrillin-1 in WT and Tsk mouse aqueous humor was cropped.

White enclosure: cropped bands shown in Fig. 5B.

WT Tsk

**Fig S7. Fibrillin-1 in mouse serum.** Full blot from which

Fig. 6C immunoblot of Fibrillin-1 in WT and Tsk mouse

serum was cropped. White enclosure: cropped bands

shown in Fig. 6C.
